# Comunicación no verbal durante la pandemia por la enfermedad de Coronavirus 2019

**DOI:** 10.21142/2523-2754-0903-2021-070

**Published:** 2021-10-06

**Authors:** César Andrés Borja-Villanueva, Luis Alexis Bernuy-Torres, Christian Esteban Gómez-Carrión, Adriana Paola Simbrón-Macera

**Affiliations:** 1 Universidad Católica Los Ángeles de Chimbote. Chimbote, Perú. abv1979@gmail.com, Universidad Católica de Los Ángeles de Chimbote Universidad Católica Los Ángeles de Chimbote Chimbote Peru abv1979@gmail.com; 2 Universidad Privada Juan Pablo II. Lima, Perú. luisbernuytorres@gmail.com, asimbronmacera@gmail.com Universidad Juan Pablo II Universidad Privada Juan Pablo II Lima Peru luisbernuytorres@gmail.com asimbronmacera@gmail.com; 3 Universidad Privada Norbert Wiener. Lima, Perú. christiangomca@gmail.com Universidad Privada Norbert Wiener Universidad Privada Norbert Wiener Lima Peru christiangomca@gmail.com

**Keywords:** comunicación no verbal, infecciones por coronavirus, COVID-19, mascarillas, sonrisa, nonverbal communication, coronavirus infections, COVID-19, masks, smiling

## Abstract

**Objetivo:**

El presente estudio tiene como objetivo explorar las características de la comunicación no verbal durante la pandemia por enfermedad de coronavirus.

**Materiales y métodos:**

Estudio exploratorio y transversal, se trabajó en la población de Lima entre 18 y 65 años, con una muestra de 391 miembros, seleccionados con muestreo por conveniencia. Se aplicó un cuestionario virtual de 9 preguntas sobre la variable comunicación no verbal y 3 preguntas sobre datos de filiación.

**Resultados:**

Los resultados de la dimensión kinésica señalan que la mayoría no aumento o disminuyó su frecuencia de gestos y expresiones faciales (51,9%) y su amplitud de sonrisa (42,7%). En la dimensión proxémica, se encontró que la mayoría disminuyó considerablemente la frecuencia de sus besos en la mejilla (74,2%), de sus besos en los labios (56%) y de la intensidad de los besos (54,5%). Para la dimensión paralingüística, se encontró que la mayoría no aumento ni disminuyó el volumen de su voz (52,4%), la velocidad al hablar y la articulación de sus palabras (57,5%).

**Conclusiones:**

La pandemia por la enfermedad del coronavirus ha producido cambios en la comunicación no verbal. El más importante se da en los besos en los labios y la mejilla, que redujeron su frecuencia e intensidad; el volumen y la velocidad de la voz no ha sufrido grandes variaciones. Los gestos, las expresiones y la sonrisa tampoco ha sufrido cambios importantes.

## Abstract (en)

**Aim:**

The present study aimed to explore the characteristics of non-verbal communication during the coronavirus disease pandemic.

**Materials and Methods:**

An exploratory cross-sectional study was carried out in a population between 18 and 65 years old from Lima. The participants were selected by convenience sampling. A virtual questionnaire of 9 questions was applied on non-verbal communication variable and 3 questions about parentage data.

**Results:**

A total of 391 individuals were included. The results of the kinesic dimension indicate that the frequency of gestures and facial expressions (51.9%) and the width of the smile (42.7%) did not change during the pandemic. In the proxemic dimension, it was found that the majority considerably decreased the frequency of kissing on the cheek (74.2%), kissing on the lips (56%) and the intensity of kissing (54.5%). For the paralinguistic dimension, the volume of the voice did not change in most individuals (52.4%) and nor did the speed of speech or articulation of words (57.5%).

**Conclusions:**

The COVID-19 pandemic has produced changes in non-verbal communication, the most important being a reduction in the frequency and intensity of kissing on the lips and cheek, while the volume and speed of the voice did not vary greatly. Neither have gestures, expressions and smiling undergone major changes.

## INTRODUCCIÓN

La comunicación se considera un proceso que consiste en la transmisión e intercambio de mensajes entre un emisor y un receptor. Las habilidades que se desarrollan dentro de la comunicación ayudan a mejorar ese intercambio, para interpretar de forma eficaz el mensaje en el sentido que pretende el emisor ^1^. La comunicación humana tiene dos formas: la comunicación verbal y la comunicación no verbal. la verbal son las palabras y la no verbal el contacto visual, las expresiones faciales, el volumen de la voz, los movimientos del cuerpo y la distancia corporal ^2^.

Entre el 60% y el 65% de los mensajes en la comunicación se emiten de forma no verbal ^3^ y están relacionados con lo verbal, ya que pueden reforzarlo, sustituirlo e incluso contradecirlo, y dar un significado distinto al mensaje. Las señales no verbales pueden ser menos susceptibles a la censura y, por lo tanto, son indicadores más confiables de lo que se está comunicando ^4^.

En el Perú, el Estado de Emergencia Nacional se decretó el 13 de marzo del 2020. Debido a ello, se dictaron las mismas medidas que los países de Asia y Europa ya habían adoptado ^5^^-^^7^. Estas trajeron como consecuencia un cambio en las comunicaciones, de pronto se pasó a la nueva normalidad de entornos virtuales ^8^^-^^10^ y mascarillas.

El distanciamiento social evita el contacto físico; por lo tanto, los saludos de manos, besos en la mejilla y los abrazos son evitados, para prevernir el contagio por secreciones ^5^^,^^6^^,^^11^. Incluso, el contacto sexual fue estigmatizado como posible agente de transmisión y contagio ^1^^,^^12^^,^^13^.

El tercio inferior del rostro transmite una gran proporción de la comunicación no verbal ^14^^)^ ; por tanto, si bien las mascarillas salvan vidas, también crean desafíos en las relaciones sociales. Las palabras por sí solas no son suficientes para comunicar actitudes, sentimientos y pensamientos que son importantes para crear vínculos emocionales entre las personas ^8^^,^^15^.

A criterio de Bernstein ^16^, para compensar el déficit de comunicación por el uso de mascarillas durante la pandemia, se acentuaron los gestos en el tercio superior del rostro, usando las cejas, los movimientos de la cabeza y la mirada. 

Para De Lima ^17^, con el uso masivo de las mascarillas, se perdió la expresividad que genera el movimiento de los órganos fonoarticuladores. Resalta que las máscaras provocan una atenuación entre 5 a 12 dB en la intensidad del sonido del habla, principalmente en las frecuencias entre 2000 y 7000 Hz. Tales frecuencias son responsables de la discriminación de varios fonemas. York ^18^ explica el concepto de la “voz enmascarada” como un uso adecuado de la pausa, la acentuación, el volumen y la emoción para superar la barrera física del uso de la mascarilla y crear una respuesta favorable del interlocutor.

Para Ribeiro ^19^, el uso de mascarillas aumentó la percepción sobre la dificultad del habla, la respiración y el esfuerzo vocal; síntomas relacionados al uso de mascarillas en actividades profesionales y esenciales. A mayor uso de las mascarillas se encontró mayor percepción del esfuerzo y la fatiga vocal. Un estudio ha demostrado que cuando los médicos usan mascarilla durante las consultas se presenta un impacto negativo en la empatía percibida por el paciente y disminuye los efectos positivos de la relación médico-paciente ^20^.

Samarasekara ^21^ encontró que el uso de mascarillas faciales y gafas de protección dificultan la comunicación no verbal con los pacientes. Rubio y García ^22^ señalan que la comunicación no verbal es importante para la interacción en el ámbito sanitario, más aún en la situación de pandemia actual.

Los trabajos sobre cómo se ha visto afectada la comunicación no verbal en esta pandemia son escasos. Por lo tanto, esta investigación tiene como objetivo explorar las características de la comunicación no verbal durante la pandemia por enfermedad del coronavirus 2019.

## MATERIALES Y MÉTODOS

La investigación es exploratoria y transversal, ejecutada entre el 26 de junio y el 26 de julio del 2020, con una población de 18 a 65 años residentes de Lima que, de acuerdo con el Instituto Nacional de Estadísticas e Informática, son 9 674 755. Se utilizó la fórmula para determinar la muestra en poblaciones infinitas con un p = 0,5 y un q = 0,5, un nivel de confianza del 95% y un error muestral del 5%, lo que da una muestra mínima de 385 personas, las cuales fueron seleccionadas mediante muestreo no probabilístico por conveniencia. Se utilizó como criterio de inclusión haber declarado su voluntad de participar en el estudio y llenar por completo el formulario, y como criterio de exclusión, que residan fuera de Lima. Participaron 405 personas, pero debido a que los encuestados podían omitir cualquier pregunta o dejar de responder la encuesta en cualquier momento, no todos respondieron el cuestionario completo y un total de 14 cuestionarios fueron excluidos de la muestra, con lo que la muestra final se redujo a 391 personas.

Se aplicó un instrumento virtual utilizando el formulario Google Forms, el cual se difundió a través de las redes sociales Facebook y WhatsApp. Fue validado por juicio de expertos aplicando el índice cuantitativo de Lawshe modificado por Tristán, con un panel de 5 profesionales con experiencia en las áreas de comunicación, odontología, psicología y sociología, cada uno con experiencia en investigación. Se remitió el cuestionario de 13 preguntas junto con la matriz de operacionalización y la matriz de consistencia de la investigación. Cada pregunta se categorizó de la siguiente manera: esencial, útil pero no esencial y no necesaria. Luego de la evaluación de contenido de cada pregunta, se descartó una de ellas por no haber consenso entre los panelistas.

El cuestionario, finalmente, consistió en 12 preguntas, las primeras 3 sobre sus datos de filiación y las otras 9 fueron preguntas cerradas, de alternativa múltiple tipo Likert, y obedecieron a las 3 dimensiones de la comunicación no verbal: kinésico, paralingüístico y proxémico ^23^, que explican la variable del estudio.

La dimensión kinésica incluye todos los movimientos que con su ejecución transmiten información, como los gestos, las posturas y las miradas. Entre ellos, son los movimientos del rostro los que más señales comunicativas transmiten. Son numerosos los gestos que se pueden producir con la nariz, las cejas, los labios y los ojos; por tanto, los movimientos faciales brindan abundante información sin emitir palabras. Son 3000 expresiones, aproximadamente, las que se pueden producir en el rostro, muchas de ellas imperceptibles por la velocidad con la que se producen, pero ricas en significados ^24^^,^^25^.

La dimensión proxémica se refiere a las distancias interpersonales y sus resultados en el entorno social, es decir, el contacto corporal. Por ende, esta dimensión se refiere al uso y la percepción que el ser humano tiene de su espacio físico, de su intimidad personal, de cómo y con quién lo utiliza. Existen acciones propias de esta dimensión como los saludos de mano, de beso, las pal-madas en la espalda, los saludos a distancia y otros ^24^^,^^25^.

La dimensión paralingüística la componen elementos que le imprimen intención y significado a la información del hablante, como el tono, la intensidad de la voz, el énfasis y las pausas ^24^^,^^25^.

El análisis de la confiabilidad del instrumento, a partir del alfa de Cronbach, para una muestra piloto de 50 participantes, dio como resultado 0,66. Para la primera dimensión fue 0,68; para la segunda, 0,85, y para la tercera, 0,63.

Los posibles sesgos de las respuestas son controlados por la propia naturaleza del tipo de instrumento, que permite una comunicación no jerárquica y libre de influencias; además, para el caso específico, el participante no necesitó vincular su correo electrónico al Google Forms, de forma que el anonimato absoluto favorece la desinhibición y disminuye la deseabilidad y ansiedad social. Así mismo, el tamaño del instrumento ha sido corto para no saturar a los participantes y las alternativas de respuestas fueron aleatorizadas en cada caso. 

El procesamiento de los datos se realizó en Microsoft Excel y el análisis estadístico en el *software* IBM-SPSS v.24. Para ello, se aplicó la prueba estadística chi cuadrado considerando la naturaleza categórica de las variables involucradas, evaluando la relación entre las tres dimensiones de la variable comunicación no verbal con el sexo, la edad y el nivel educativo. En estas pruebas se consideró el nivel de significancia de 0,05.

El cuestionario contaba en su encabezado con el consentimiento, el cual incluía una breve descripción de los objetivos, la indicación de su naturaleza anónima, así como una invitación a participar con la opción de responder “sí” o “no”. Se tomó en consideración los cuestionarios con respuesta afirmativa de participación. Se aplicaron los principios éticos de la declaración de Helsinki y se contó con la aprobación del Comité de Ética de la Universidad Privada Juan Pablo II, mediante Resolución N.º 005-2020-DI/CE.

## RESULTADOS

En la Tabla 1, con respecto a la dimensión kinésica, se observa que 8 de cada 100 participantes aumentaron considerablemente la frecuencia de sus gestos y expresiones faciales, mientras que 1 de cada 5 lo aumentó y la mitad de la muestra (51,9%) ni aumentaron ni disminuyeron sus gestos y expresiones faciales.

En cuanto a la sonrisa, la suma de las alternativas D y E, es decir, el 47,1% de los participantes, disminuyó su frecuencia durante la pandemia. En cuanto a la amplitud de la sonrisa, el 5,9% La disminuyó considerablemente y el 33,2% solo la disminuyó. 

En cuanto a los resultados correspondientes a la dimensión proxémica, la Tabla 1 evidencia que el 74,2% de la muestra disminuyó considerablemente la frecuencia de sus saludos con beso en la mejilla y el 14,3% la disminuyó, resultados que suman 88 personas de cada 100. Más de la mitad de los participantes (56%) disminuyó considerablemente la frecuencia de sus besos en los labios y también más de la mitad (54,5%) disminuyó considerablemente la intensidad de sus besos.

En cuanto a la dimensión paralingüística, la Tabla 1 nos muestra que la quinta parte (20,5%) de los participantes disminuyó considerablemente su velocidad al hablar y la articulación de sus palabras durante la pandemia. Por otra parte, el 19,2% disminuyó el volumen de su voz; sin embargo, un 21,7% también aumentó dicho volumen. En cuanto a los bostezos, estos habrían aumentado en un 40,2% y aumentado considerablemente en un 17,6%.

**Tabla 1:** Caracterización de la comunicación no verbal

ÍTEM	A. Aumentaron considerablemente		B. Aumentaron		C. Ni aumentaron ni disminuyeron		D. Disminuyeron		E. Disminuyeron considerablemente		Total	
	N.º	%	N.º	%	N.º	%	N.º	%	N.º.	%	N.º	%
Dimensión kinésica												
La frecuencia de sus gestos y expresiones faciales durante la pandemia	32	8,2	80	20,5	203	51,9	57	14,6	19	4,9	391	100,0
La frecuencia con la que usted sonríe en cuarentena	19	4,9	43	11,0	145	37,1	147	37,6	37	9,5	391	100,0
La amplitud de su sonrisa en la cuarentena	19	4,9	52	13,3	167	42,7	130	33,2	23	5,9	391	100,0
Dimensión proxémica												
La frecuencia de sus saludos con beso en la mejilla en la pandemia por coronavirus	5	1,3	4	1,0	36	9,2	56	14,3	290	74,2	391	100,0
La frecuencia de sus besos en los labios en la pandemia por coronavirus	6	1,5	10	2,6	97	24,8	59	15,1	219	56,0	391	100,0
La intensidad de sus besos en la pandemia por coronavirus	6	1,5	13	3,3	99	25,3	60	15,3	213	54,5	391	100,0
Dimensión paralingüística												
El volumen de su voz durante la pandemia	19	4,9	85	21,7	205	52,4	75	19,2	7	1,8	391	100,0
La velocidad de su habla y de la articulación de sus palabras durante la pandemia	8	2,0	20	5,1	225	57,5	58	14,8	80	20,5	391	100,0
La frecuencia de silbidos durante la pandemia	47	12,0	124	31,7	114	29,2	71	18,2	35	9,0	391	100,0

Fuente: Elaboración propia

En la Tabla 2 se puede observar la caracterización de la comunicación no verbal de acuerdo con el sexo. En el caso de la frecuencia de los gestos y las expresiones, los resultados han sido similares, mientras que, en cuanto a la frecuencia de las sonrisas, sí hay una mayor disminución en el sexo femenino (un 50% es la suma de las que disminuyeron y disminuyeron considerablemente su frecuencia de sonrisas) que en el masculino, donde la mayoría conservó su frecuencia (47,4%). La amplitud de la sonrisa en el sexo masculino, en su mayoría, se conservó (55,7%), mientras que en el sexo femenino el 41,5% la disminuyó o la disminuyó considerablemente. 

**Tabla 2:** Caracterización de la comunicación no verbal de acuerdo al sexo

Sexo	A. Aumentaron considerablemente		B. Aumentaron		C. Ni aumentaron ni disminuyeron		D. Disminuyeron		E. Disminuyeron considerablemente		Total		Ítem	p
	N.º	%	N.º	%	N.º	%	N.º	%	N.º	%	N.º	%		
Dimensión kinésica					
Femenino	26	8,8%	58	19,7%	155	52,7%	41	13,9%	14	4,8%	294	100,0%	La frecuencia de sus gestos y expresiones faciales durante la pandemia por coronavirus	0,679
Masculino	6	6,2%	22	22,7%	48	49,5%	16	16,5%	5	5,2%	97	100,0%		
Femenino	14	4,8%	34	11,6%	99	33,7%	116	39,5%	31	10,5%	294	100,0%	La frecuencia con la que usted sonríe durante la pandemia por coronavirus	0,116
Masculino	5	5,2%	9	9,3%	46	47,4%	31	32,0%	6	6,2%	97	100,0%		
Femenino	15	5,1%	44	15,0%	113	38,4%	107	36,4%	15	5,1%	294	100,0%	La amplitud de su sonrisa durante la pandemia por coronavirus	0,81
Masculino	4	4,1%	8	8,2%	54	55,7%	23	23,7%	8	8,2%	97	100,0%		
Dimensión proxémica														
Femenino	3	1,0%	4	1,4%	24	8,2%	44	15,0%	219	74,5%	294	100,0%	La frecuencia de sus saludos con beso en la mejilla durante la pandemia por coronavirus	0,693
Masculino	2	2,1%	0	0,0%	12	12,4%	12	12,4%	71	73,2%	97	100,0%		
Femenino	4	1,4%	6	2,0%	72	24,5%	49	16,7%	163	55,4%	294	100,0%	La frecuencia de sus besos en los labios durante la pandemia por coronavirus	0,897
Masculino	2	2,1%	4	4,1%	25	25,8%	10	10,3%	56	57,7%	97	100,0%		
Femenino	4	1,4%	10	3,4%	74	25,2%	47	16,0%	159	54,1%	294	100,0%	La intensidad de sus besos durante la pandemia por coronavirus	0,927
Masculino	2	2,1%	3	3,1%	25	25,8%	13	13,4%	54	55,7%	97	100,0%		
Dimensión paralingüística														
Femenino	18	6,1%	67	22,8%	148	50,3%	56	19,0%	5	1,7%	294	100,0%	El volumen de su voz durante la pandemia por coronavirus	0,147
Masculino	1	1,0%	18	18,6%	57	58,8%	19	19,6%	2	2,1%	97	100,0%		
Femenino	7	2,4%	13	4,4%	175	59,5%	40	13,6%	59	20,1%	294	100,0%	La velocidad de su habla y de la articulación de sus palabras durante la pandemia por coronavirus	0,386
Masculino	1	1,0%	7	7,2%	50	51,5%	18	18,6%	21	21,6%	97	100,0%		
Femenino	40	13,6%	96	32,7%	80	27,2%	49	16,7%	29	9,9%	294	100,0%	La frecuencia de silbidos durante la pandemia por coronavirus	0,459
Masculino	7	7,2%	28	28,9%	34	35,1%	22	22,7%	6	6,2%	97	100,0%		

Fuente: Elaboración propia

En la Tabla 2 se observa un comportamiento similar entre los sexos, tanto para la frecuencia de los besos en la mejilla, como para los besos en los labios y la intensidad de los besos. En los tres casos, ambos sexos disminuyeron considerablemente estos comportamientos. También podemos visualizar en la Tabla 2 que ambos sexos tuvieron un comportamiento similar para el volumen de la voz y la velocidad del habla y la articulación de palabras: el 50,3% de la muestra femenina y el 50,8% de la masculina mantuvieron su volumen de voz durante la pandemia, mientras que el 59,5% de la muestra femenina y el 51,5% de la masculina mantuvieron la velocidad de su habla y articulación de palabras. 

En la Tabla 3 se puede observar la caracterización de la comunicación no verbal de acuerdo con la edad. En la frecuencia de gestos y expresiones hay un resultado similar entre los tres grupos etarios, mayoritariamente, ni aumentaron ni disminuyeron su frecuencia. Lo mismo sucede en cuanto a la frecuencia y amplitud de la sonrisa, donde tuvieron un comportamiento similar entre ellos, pues en su mayoría respondieron que mantuvieron o disminuyeron su frecuencia y amplitud de sonrisas. 

**Tabla 3:** Caracterización de la comunicación no verbal de acuerdo al grupo etario.

Edad	A. Aumentaron considerablemente		B. Aumentaron		C. Ni aumentaron ni disminuyeron		D. Disminuyeron		E. Disminuyeron considerablemente		Total		Ítem	P
	N.º	%	N.º	%	N.º	%	N.º	%	N.º	%	N.º	%		
Dimensión kinésica					
18-30 años	22	7,6%	55	19,0%	153	52,9%	45	15,6%	14	4,8%	289	100,0%	La frecuencia de sus gestos y expresiones faciales durante la pandemia por coronavirus	0,17
31-45 años	6	9,8%	13	21,3%	29	47,5%	10	16,4%	3	4,9%	61	100,0%		
46-65 años	4	9,8%	12	29,3%	21	51,2%	2	4,9%	2	4,9%	41	100,0%		
18-30 años	16	5,5%	31	10,7%	106	36,7%	108	37,4%	28	9,7%	289	100,0%	La frecuencia con la que usted sonríe durante la pandemia por coronavirus	0,816
31-45 años	2	3,3%	7	11,5%	24	39,3%	25	41,0%	3	4,9%	61	100,0%		
46-65 años	1	2,4%	5	12,2%	15	36,6%	14	34,1%	6	14,6%	41	100,0%		
18-30 años	15	5,2%	40	13,8%	126	43,6%	91	31,5%	17	5,9%	289	100,0%	La amplitud de su sonrisa durante la pandemia por coronavirus	0,299
31-45 años	3	4,9%	7	11,5%	27	44,3%	21	34,4%	3	4,9%	61	100,0%		
46-65 años	1	2,4%	5	12,2%	14	34,1%	18	43,9%	3	7,3%	41	100,0%		
Dimensión proxémica														
18-30 años	4	1,4%	2	0,7%	32	11,1%	41	14,2%	210	72,7%	289	100,0%	La frecuencia de sus saludos con beso en la mejilla durante la pandemia por coronavirus	0,373
31-45 años	0	0,0%	2	3,3%	1	1,6%	9	14,8%	49	80,3%	61	100,0%		
46-65 años	1	2,4%	0	0,0%	3	7,3%	6	14,6%	31	75,6%	41	100,0%		
18-30 años	4	1,4%	8	2,8%	66	22,8%	38	13,1%	173	59,9%	289	100,0%	La frecuencia de sus besos en los labios durante la pandemia por coronavirus	0.76
31-45 años	0	0,0%	2	3,3%	19	31,1%	14	23,0%	26	42,6%	61	100,0%		
46-65 años	2	4,9%	0	0,0%	12	29,3%	7	17,1%	20	48,8%	41	100,0%		
18-30 años	4	1,4%	11	3,8%	69	23,9%	46	15,9%	159	55,0%	289	100,0%	La intensidad de sus besos en la pandemia por coronavirus	0,414
31-45 años	0	0,0%	2	3,3%	21	34,4%	9	14,8%	29	47,5%	61	100,0%		
46-65 años	2	4,9%	0	0,0%	9	22,0%	5	12,2%	25	61,0%	41	100,0%		
Dimensión paralingüística														
18-30 años	11	3,8%	61	21,1%	153	52,9%	58	20,1%	6	2,1%	289	100,0%	El volumen de su voz durante la pandemia por coronavirus	0,18
31-45 años	2	3,3%	19	31,1%	32	52,5%	8	13,1%	0	0,0%	61	100,0%		
46-65 años	6	14,6%	5	12,2%	20	48,8%	9	22,0%	1	2,4%	41	100,0%		
18-30 años	7	2,4%	19	6,6%	152	52,6%	40	13,8%	71	24,6%	289	100,0%	La velocidad de su habla y de la articulación de sus palabras durante la pandemia	
31-45 años	0	0,0%	1	1,6%	43	70,5%	12	19,7%	5	8,2%	61	100,0%		
46-65 años	1	2,4%	0	0,0%	30	73,2%	6	14,6%	4	9,8%	41	100,0%		
18-30 años	30	10,4%	91	31,5%	97	33,6%	50	17,3%	21	7,3%	289	100,0%	La frecuencia de silbidos durante la pandemia por coronavirus	0,257
31-45 años	8	13,1%	24	39,3%	14	23,0%	8	13,1%	7	11,5%	61	100,0%		
46-65 años	9	22,0%	9	22,0%	3	7,3%	13	31,7%	7	17,1%	41	100,0%		

Fuente: Elaboración propia

Con respecto a la frecuencia de los saludos con besos en la mejilla, fue similar entre los grupos etarios, y estos tuvieron una disminución considerable superior al 70%. Sobre los besos en los labios también respondieron que la disminuyeron considerablemente. El volumen de la voz tuvo un comportamiento similar en los 3 grupos, pues en su mayoría ni lo aumentaron ni lo disminuyeron. 

En cuanto a la velocidad del habla y de la articulación de las palabras, los grupos etarios de 31-45 años y 46-65 años mantuvieron su comportamiento como antes de la pandemia en porcentajes superiores al 70%; para el caso del grupo etario de 18-30 años, el porcentaje fue del 52,6%. 

Con respecto al número de silbidos, los resultados por grupos etarios son distintos; lo más resaltante es que solo el 7,3% del grupo de 45-65 años mantuvo el mismo comportamiento de antes de la pandemia.

En la Tabla 4 se puede observar la caracterización de la comunicación no verbal de acuerdo con el nivel educativo. La frecuencia de los gestos y las expresiones en tres niveles educativos (secundaria, superior técnica y superior universitaria), mayoritariamente, no aumentó ni disminuyó; sin embargo, resalta el 35,5% del nivel superior técnico, que aumentó su frecuencia de gestos y expresiones. En cuanto a la frecuencia y la amplitud de las sonrisas, también han tenido un comportamiento similar, en su mayoría no cambiaron su comportamiento en pandemia o lo disminuyeron. 

**Tabla 4:** Caracterización de la comunicación no verbal de acuerdo al nivel educativo

NIVEL EDUCATIVO	A. Aumentaron considerablemente		B. Aumentaron		C. Ni aumentaron ni disminuyeron		D. Disminuyeron		E. Disminuyeron considerablemente		Total		Pregunta	P
	N.º	%	N.º	%	N.º	%	N.º	%	N.º	%	N.º	%				
Dimensión kinésica														
Primaria	0	0,0	1	25,0	2	50,0	0	0,0	1	25,0	4	100,0%	La frecuencia de sus gestos y expresiones faciales durante la pandemia por coronavirus	0,292
Secundaria	6	6,2	24	24,2	53	53,5	14	14,1	2	2,0	99	100,0%		
Superior técnico	2	3,2	22	35,5	29	46,8	7	11,3	2	3,2	62	100,0%		
Superior universitario	24	10,6	33	14,6	119	52,7	36	15,9	14	6,2	226	100,0%		
Primaria	2	50,0	0	0,0	1	25,0	1	25,0	0	0,0	4	100,0%	La frecuencia con la que usted sonríe durante la pandemia por coronavirus	0,219
Secundaria	6	6,1	11	11,1	41	41,4	30	30,3	11	11,1	99	100,0%		
Superior técnico	4	6,5	7	11,3	23	37,1	23	37,1	5	8,1	62	100,0%		
Superior universitario	7	3,1	25	11,1	80	35,4	93	41,2	21	9,3	226	100,0%		
Primaria	2	50,0	1	25,0	1	25,0	0	0,0	0	0,0	4	100,0%	La amplitud de su sonrisa durante la pandemia por coronavirus	0,02
Secundaria	7	7,1	9	9,1	54	54,5	24	24,2	5	5,1	99	100,0%		
Superior técnico	3	4,8	7	11,3	26	41,9	23	37,1	3	4,8	62	100,0%		
Superior universitario	7	3,1	35	15,5	86	38,1	83	36,7	15	6,6	226	100,0%		
Dimensión proxémica														
Primaria	1	25,0	0	0,0	1	25,0	1	25,0	1	25,0	4	100,0%	La frecuencia de sus saludos con beso en la mejilla durante la pandemia por coronavirus	0,00
Secundaria	2	2,0	3	3,0	14	14,1	18	18,2	62	62,6	99	100,0%		
Superior técnico	1	1,6	1	1,6	7	11,3	9	14,5	44	71,0	62	100,0%		
Superior universitario	1	0,4	0	0,0	14	6,2	28	12,4	183	81,0	226	100,0%		
Primaria	1	25,0	0	0,0	0	0,0	2	50,0	1	25,0	4	100,0%	La frecuencia de sus besos en los labios durante la pandemia por coronavirus	0,637
Secundaria	2	2,0	3	3,0	26	26,3	15	15,2	53	53,5	99	100,0%		
Superior técnico	1	1,6	1	1,6	15	24,2	11	17,7	34	54,8	62	100,0%		
Superior universitario	2	0,9	6	2,7	56	24,8	31	13,7	131	58,0	226	100,0%		
Primaria	1	25,0	0	0,0	0	0,0	2	50,0	1	25,0	4	100,0%	La intensidad de sus besos durante la pandemia por coronavirus	0,561
Secundaria	1	1,0	3	3,0	29	29,3	17	17,2	49	49,5	99	100,0%		
Superior técnico	1	1,6	2	3,2	15	24,2	9	14,5	35	56,5	62	100,0%		
Superior universitario	3	1,3	8	3,5	55	24,3	32	14,2	128	56,6	226	100,0%		
Dimensión paralingüística														
Primaria	0	0,0	0	0,0	4	100,0	0	0,0	0	0,0	4	100,0%	El volumen de su voz durante la pandemia por coronavirus	0,386
Secundaria	2	2,0	18	18,2	56	56,6	19	19,2	4	4,0	99	100,0%		
Superior técnico	4	6,5	13	21,0	31	50,0	13	21,0	1	1,6	62	100,0%		
Superior universitario	13	5,8	54	23,9	114	50,4	43	19,0	2	0,9	226	100,0%		
Primaria	0	0,0	0	0,0	2	50,0	2	50,0	0	0,0	4	100,0%	La velocidad de su habla y de la articulación de sus palabras durante la pandemia por coronavirus	0,452
Secundaria	2	2,0	5	5,1	57	57,6	14	14,1	21	21,2	99	100,0%		
Superior técnico	0	0,0	4	6,5	30	48,4	12	19,4	16	25,8	62	100,0%		
Superior universitario	6	2,7	11	4,9	136	60,2	30	13,3	43	19,0	226	100,0%		
Primaria	2	50,0	2	50,0	0	0,0	0	0,0	0	0,0	4	100,0%	La frecuencia de silbidos durante la pandemia por coronavirus	0,393
Secundaria	10	10,1	29	29,3	37	37,4	18	18,2	5	5,1	99	100,0%		
Superior técnico	7	11,3	14	22,6	25	40,3	11	17,7	5	8,1	62	100,0%		
Superior universitario	28	12,4	79	35,0	52	23,0	42	18,6	25	11,1	226	100,0%		

Fuente: Elaboración propia

Para la frecuencia de besos en la mejilla y en los labios, así como la intensidad de los mismos en los niveles secundario, superior técnico y superior universitario disminuyeron considerablemente. Para el volumen y la velocidad del habla y de la articulación de palabras, en todos los niveles educativos, generalmente no aumentaron ni disminuyeron su comportamiento. Lo mismo se cumple para el caso de la frecuencia de silbidos, con excepción del nivel educativo superior universitario y primaria, que sí aumentaron su frecuencia.

## DISCUSIÓN

Con respecto al volumen de la voz durante la pandemia, el presente estudio muestra que la población ha mantenido su mismo volumen de voz (52,4%) o lo ha disminuido (21%); resultado contrario a lo que sugiere Mheidly ^26^, que aumentar el volumen de voz es el ajuste necesario para superar la amortiguación del sonido por el uso de las mascarillas. Heider ^27^, en su estudio sobre la prevalencia de trastornos de la voz en los trabajadores de la salud en la era del enmascaramiento universal COVID-19, encuentra que el 21,56% de los participantes autopercibieron problemas leves con su voz y el 11%, síntomas moderados a graves.

Sobre la velocidad del habla y la articulación de las palabras, el presente estudio encontró que el 57,5% ha mantenido la misma velocidad y que el 35,3% ha disminuido la velocidad de su habla y la articulación de palabras, resultados que no tienen semejanza con la sugerencia que plantean Mheidly ^26^, Ringle y Bruce ^28^ de disminuir la velocidad del habla para superar la barrera física de las mascarillas faciales. 

Berger ^29^, en su estudio sobre el fallo de comunicación, encontró que, ante la imposibilidad de comunicarse de forma efectiva, los participantes demostraron aumentos significativos en la intensidad vocal y disminuciones en la velocidad del habla. Lo mismo señalan Rubio y García ^22^, acerca de que el uso de equipos de protección, como las mascarillas, genera distorsiones en la modulación de la voz, incrementa el tono y distorsiona las emociones del mensaje transmitido. 

Mheidly ^26^ señala que, en esta nueva normalidad, una de las medidas para afrontar las dificultades de la comunicación sería enfatizar las expresiones faciales, los movimientos corporales y los mensajes oculares, que pueden apoyar o sustituir la comunicación verbal, situación que no se refleja en los resultados del presente estudio, en el que la mitad de los participantes (51,3%) no aumentó ni disminuyó su frecuencia de gestos y expresiones faciales durante la pandemia, solo un 20,8% los aumentó y el 8,2% los aumentó considerablemente. 

A criterio de Cela ^30^, en su trabajo sobre los efectos del uso de las mascarillas en la comunicación, el uso de estas como elemento de protección primaria también produce una barrera física que dificulta la comunicación no verbal. Esto guarda relación con los resultados del presente estudio, ya que se ha encontrado que los participantes disminuyeron la frecuencia de sus sonrisas un 37,6% y la disminuyeron considerablemente un 9,5%, lo que suma un total del 47,1%.

El resultado de la presente investigación sobre la amplitud de la sonrisa durante la pandemia coincide con lo señalado por Cela ^30^, cuando indica que las sonrisas, los movimientos de boca, las muecas y los gestos ya no están para dar respaldo a nuestras palabras y enunciados; lo mismo para Rubio y García ^22^, quienes señalan que el uso de las mascarillas hace imposible observar las sonrisas y que las gafas o los protectores faciales dificultan “la sonrisa con los ojos”. 

Matschke ^31^, en su trabajo sobre los saludos de manos y de besos en una población de Alemania, encontró que, antes de la pandemia, el 30% usaba el beso en la mejilla como saludo y, durante la pandemia, este porcentaje disminuyó hasta el 1%, lo que es coherente con los resultados encontrados en el presente estudio, el cual señala que un 74,2% disminuyó considerablemente sus saludos con beso en la mejilla y un 14,3% los disminuyó. 

Durante la pandemia, el Departamento de Salud de Nueva York ^32^ recomendó evitar los besos en los labios, medida que guarda relación con los resultados del presente estudio, en el que más de la mitad de los participantes disminuyeron considerablemente (56%) sus besos en los labios y más de la mitad (54,5%) los disminuyó considerablemente.

En cuanto a las limitaciones del estudio, se evaluó la confiabilidad del instrumento mediante el análisis de consistencia en una sola administración (muestra piloto), pero no se evaluó la estabilidad temporal de los resultados (en dos momentos), por lo que, dada la novedad del instrumento, es recomendable valorar la estabilidad temporal en futuros estudios. Una segunda limitación corresponde al contexto de virtualidad en la cual se aplicó el instrumento; debido a ello, los participantes no contaron con indicaciones verbales o previa capacitación por parte de los investigadores para el llenado del instrumento.

## CONCLUSIONES

La pandemia por la enfermedad del coronavirus ha producido algunos cambios en la comunicación no verbal, que afectan sobre todo su dimensión proxémica, por lo que ha disminuido la frecuencia e intensidad de los besos en los labios y las mejillas. La dimensión paralingüística no ha sufrido variación, el volumen de la voz no cambió y tampoco la velocidad del habla. Para la dimensión kinésica, tampoco se han reportado cambios importantes, pues la frecuencia de los gestos y las expresiones, así como la frecuencia de las sonrisas y su amplitud no disminuyeron ni aumentaron durante la pandemia.

## Fuente de financiamiento:

Autofinanciado
